# A sandponics comparative study investigating different sand media based integrated aqua vegeculture systems using desalinated water

**DOI:** 10.1038/s41598-022-15291-7

**Published:** 2022-06-30

**Authors:** Hani Sewilam, Fahad Kimera, Peter Nasr, Mahmoud Dawood

**Affiliations:** 1 UNESCO Chair in Hydrological Changes and Water Resources Management, RWTH Aachen University, Aachen, Germany; 2 Center for Applied Research on the Environment and Sustainability (CARES), School of Science and Engineering, The American University in Cairo, AUC Avenue, P.O. Box 74, New Cairo, 11835 Egypt; 3 Research Consultant and Adjunct Faculty, Center for Applied Research On the Environment and Sustainability (CARES), School of Science and Engineering, The American University in Cairo, AUC Avenue, P.O. Box 74, New Cairo, 11835 Egypt; 4 Department of Animal Production, Faculty of Agriculture, Kafrelsheikh University, Kafrelsheikh, 33516 Egypt

**Keywords:** Plant sciences, Climate sciences, Ecology, Environmental sciences

## Abstract

This study investigated the utilization of fish effluents as irrigation water and nutrient sources to close the crop yield gap and increase Swiss chard productivity in a closed-loop sandponics system. The experiment was operated using desalinated water from a Reverse Osmosis plant. The study followed a completely randomized design with four variants, i.e., an aquaponic system (T1) and three sandponics systems; October (T2), Benu Suef (T3) and Fayoum (T4). Results indicated that T2 and T4 significantly recorded the highest plant heights in all cuts. The number of leaves per plant decreased with the increase in cut number. Leaf area and chlorophyll was significantly different between the treatments. T1 significantly had low biomass yields in cuts one and two, almost 40% less than T3 and T4. The various systems efficiently minimized water consumption ranging from 1.5 to 1.96 L/m^2^/day. The crop protein content ranged from 11.84 to 18.72 mg/100 g dry weight. Mineral composition in cut one was significantly higher compared to cuts two and three. The study recommends a novel technique for increasing crop production using fish effluents under sandponics systems while increasing water and fertilizer efficiency to close the crop yield gap.

## Introduction

Food insecurity has been defined by the Food and Agriculture Organization (FAO) as the lack of a sustainable amount of nutritious and safe food to support development, growth, and lead a healthy and active lifestyle^1^. Globally, nearly one in ten people were exposed to severe levels of food insecurity in 2019. The latest estimates indicate that over 8.8% of the world’s population suffers from hunger. This implies that the number of food-insecure individuals increased by more than 10 million people over the past year and by almost 60 million in the past five years^2^. Rapid industrialization and urbanization have set a substantial drawback on conventional farming by increasing greenhouse gas emissions, negatively affecting agricultural production and shrinking the available cultivable land. To ensure a secure food supply to the world’s constantly growing population, there is a dire need to develop other sustainable food growing techniques that are more productive, efficient, and environmental friendly.

Urban farming techniques such as greenhouse hydroponics production, aquaponics, sandponics, and many more are among the most efficient solutions. They feature soilless cultivation of crops, less freshwater usage with an increased crop yield per area, and most importantly, they produce healthy and chemical-free nutrient-diverse foods^3^. Aquaponics (AP), the cultivation of plants and fish together in a recirculating, manufactured ecosystem that uses natural bacterial cycles to utilize fish waste as nutrition for plants^4^. Aquaponics can be a potential solution for huge global challenges, including water pollution, water scarcity, long food transportation mileages and high energy use, and most importantly, food security and malnutrition. As cited in Li et al.^5^, AP is especially promising for urban areas to meet the additional food demands because of higher crop yields per area^6^.

On the other hand, sandponics (SP), which is also referred to as an Integrated Aqua-Vegeculture system (IAVS) is an aquaponic-related growing technique for cultivating plants that utilize sand as a primary medium for mechanical filtration, biofilter, and as well growing media for crops. It is a promising sustainable production option for several crops, including vegetables, vines, and fruits. The system can easily be applied since sand is readily available in most areas, can be easily sterilized, versatile, easily recycled, and cheaper than soil. This makes SP a more efficient, affordable, and low-risk technology. However, some limitations to the SP system need to be addressed. They include operators requiring specialized training, crop nutritional deficiencies due to insufficient fertilizers, finding suitable sand for crops that require cooler climates, and expensive heated systems. Most importantly, very few works in the literature report the system's functionality since it’s not yet a commonly practiced technique^7^. The system is tailored to enhance productivity by enabling year-round crop organic production within a controlled environment^8^. Relying solely on such farming systems to solve the food security issue may not be entirely sufficient. However, SP can produce healthy local crops to support a healthy urban/peri-urban lifestyle and eventually build a giant leap towards more nutritious and more food secure communities^9^.

The nutrient composition of water effluent in SP plays a major role in the system's overall performance. The fish wastes contain nitrogen which is presented mainly as total ammonia. Total ammonia consists of both ammonium (NH4^+^) and ammonia (NH_3_); both undergo the nitrification process to oxidize to nitrate (NO_3_^−^) Nitrogen^10^. The amount of ammonia produced in the system depends on several factors such as fish biomass, fish size, and the amount/nature of food fed to the fish. Environmental changes like water temperature, salinity, and oxygen levels can also affect all system components' activities and growth, including microbes, fish, and plants^11^.

Swiss chard Bright lights (*Beta vulgaris* subsp. *cicla*) is a multi-colored leafy plant first recorded in the Canary Islands and could be historically traced back to 350 BC^12^. The plant is also known for a variety of other names, where a popular name in Australia is “Silver Beet”; a location where it is more preferred than regular spinach. The plant is prized for its high yield and high nutritional value that it was the subject of a 13-week study that is geared towards selecting candidate cultivars for space applications as a salad crop for manned missions; the selection was not only based on high yield but also the sensory properties and growth conditions and suitability for vertical farming^13^. Swiss chard (*Beta vulgaris*) requires well-aerated crumbly soils with an ample supply of organic matter to retain adequate moisture levels. It would be sensitive to damping-off effects if the soil is not well-drained or properly ventilated^14^.

According to the US Department of Agriculture nutrient analysis, a cup containing a 36-g amount of cooked Swiss Chard has 18 mg of Calcium (Ca), 0.65 mg of Iron (Fe), 136 mg of Potassium (K), 10.8 mg of Vitamin C, 5 mg of folate, 298 mg of Vitamin K, 29 mg of Magnesium (Mg), 17 mg of Phosphorus (P), 110 mg of Vitamin A and 0.68 mg of Vitamin E. Due to the high folate content within Swiss chard, research has been conducted, making it a filler ingredient in bread making to increase its nutritional makeup. They fortified 20 g of Swiss chard per 100 g of bread increasing its folate content from 19.9 to 57.9 mg in white bread and from 37.4 to 75.5 mg in whole grain bread^15^.

There is very little or no scientific literature about growing crops in sandponics systems hence, creating so many questions related to the operation, functionality, optimization, sand suitability, and system productivity. The current study's objective was to investigate the productivity of different IAVS (Integrated Aqua-Vegeculture system) sand types and quality from various locations in Egypt on the performance, growth, and yield of Swiss chard crop compared to the deep-water culture aquaponics system.

## Materials and methods

### Study site

The study was conducted at the Center for Applied Research on the Environment and Sustainability (CARES) at The American University in Cairo, New Cairo, Egypt (30°01′11.7″N 31°29′59.8″E) from 12/Nov/2019 until 31st/March/2020. The experiment was carried out in a greenhouse-controlled environment with temperatures ranging from 18 to 23 °C and relative humidity between 60 and 70% during the growing period.

### Experimental design

**Figure 1:**
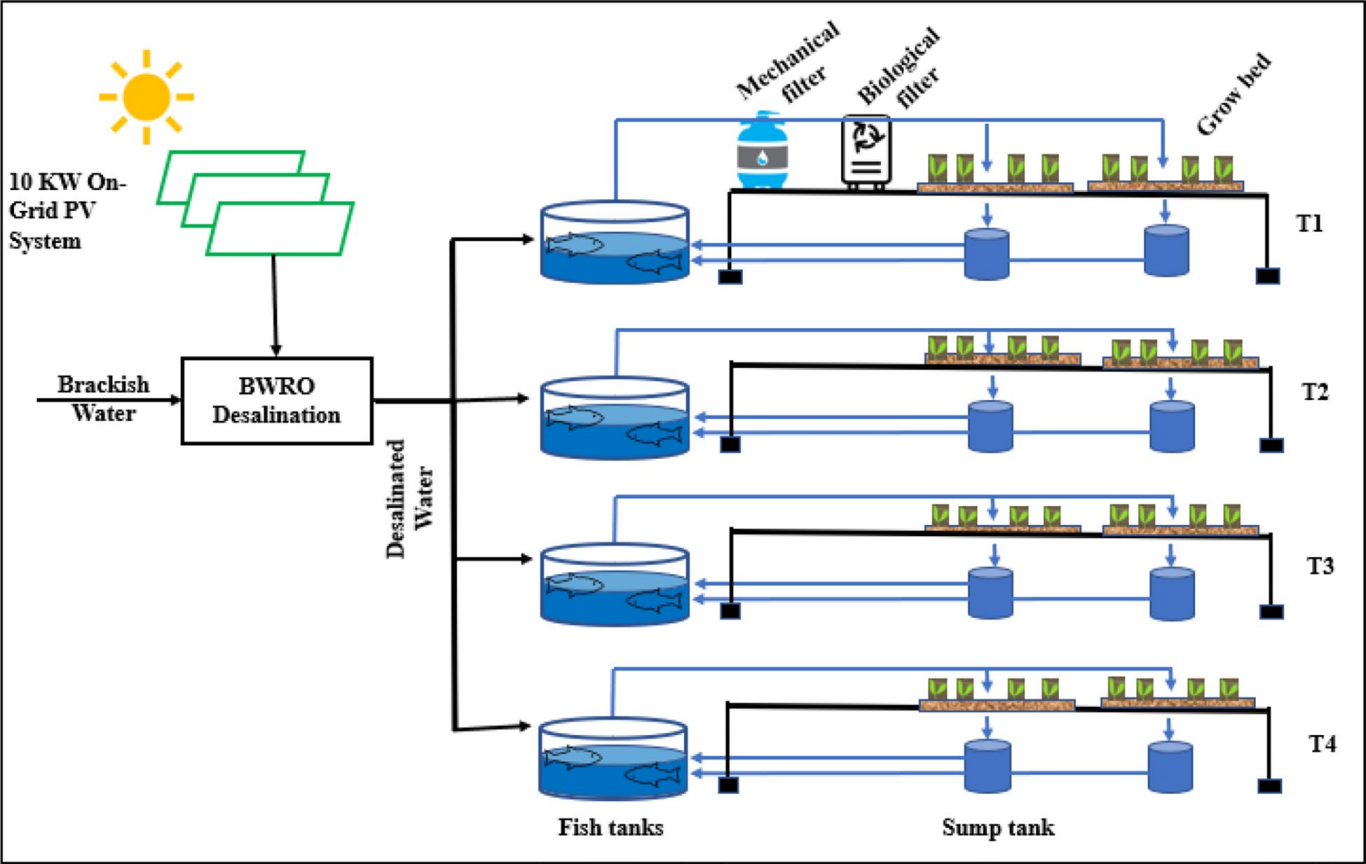
Schematic Integrated model design. *T1* Deep water culture system without sand, *T2* Sandponics system with sand from October, *T3* Sandponics system with sand from Beni suef, *T4* Sandponics system with sand from Fayoum.

The proposed design starts by treating brackish water using RO membrane separation technology, powered by an on-grid 10 kW photovoltaic solar panel as shown in Fig. 1. The permeate (freshwater) from the RO facility is directed to the aquaculture units of capacity of 1 m^3^, where the fish effluents are used as irrigation water and as the sole source of fertilizers for the crops.

The study followed a completely randomized design with four variants, i.e., an aquaponic deep-water culture system (T1) and three sandponics systems (T2–T4). The three sandponics systems were established with different sand collected from different sand locations in Egypt during the period between September and October 2019.

Initially, an exploratory field trip was set to six different locations in Egypt to collect sand samples for lab analysis aimed at sourcing the most suitable sand for the system under study with regards to both the physical and chemical parameters. These areas include Ismailia Governorate; 30°34′55.2″N 31°50′08.1″E, 6th October governorate; 29°54′49.8″N 31°05′51.5″E, Benu Suef governorate; 28°53′18.4″N 30°45′12.9″E, Al-Minya governorate; 28.725799, 30.630305, and two sites from Fayoum governorate; 29°05′07.4″N 30°49′39.9″E.

From the six locations in Egypt, preliminary sand analysis was carried out, and sand samples were also collected for both physical and chemical lab analysis at the Soil and Water Lab at the Agricultural Research Center in Dokki, Egypt. Following a thorough technical, field, mechanical, and lab chemical evaluation of the six sand samples from six locations, three sand locations/types were selected for experimentation that seemed fit and suitable for the current study. The criteria parameters for the shortlisting of sand included water retention potential of the sand by the percolation process, testing the carbonates level in the soil, the turbidity of the sand, porosity percentage and drainage potential of the sand. The three locations included 6th October (T2), Benu Suef (T3), and Fayoum site 2 (T4). In the second week of November 2019, ten cubic meter tracks of sand from the three above locations were set to collect sand from these areas to the research facility at CARES where the experiment was carried out.

The study was carried out with two systems/setups, i.e., an aquaponic Deep Water Culture (DWC) and SP systems. The DWC model comprises a 1 m^3^ fish tank, a settlement tank, a mechanical filter, a biological filter, three grow beds, and a drainage tank. This system being the most practiced aquaponics technique was considered as the control. Fish effluent water flowed from the fish tank to the settlement tank to filter big solid wastes through the mechanical filter to remove the smaller solid wastes and the biological filter for the nitrification process. Then filtered water continues to the grow beds, where overflow drains into the drainage tank and back to the fish tank in a closed system.

On the other hand, the variable in the three IAVS systems is the sand source. This system comprises three independent set-ups: a 1 m^3^ fish tank, three grow beds, and a drainage tank. Fish effluents flowed from the fish tank directly to the sand grow beds where water was supplied through irrigation drip lines using diaghram emitters connected with valves to ensure uniformity of water application to each grow bed.

All the fish tanks were installed with the same fish stock size of 30 Nile tilapia (*Oreochromis niloticus*) from an existing fish stock at the research center with an average initial weight of 244 g and the same amount of water, initially 850L per tank. The fish was sourced from an already existing aquaponics system at the research center to avoid any transportation stress effects and related shocks on the small fish, leading to a lot of mortality cases. The fish were fed 3–4 times daily with commercial pellets containing 30% proteins, 5% crude lipid, 6% crude fiber, 13% Ash, and 9% moisture content supplied by Skretting Egypt. The feeding pattern and frequency were according to the fish body biomass percentage of 2–3% depending on the growth stage and upon reaching satiation.

### Desalination

The experiment was entirely run with desalinated water produced from a desalination facility at the center. The desalination technology used was Reverse Osmosis (RO); in batch mode; using a Sea Water Pump with Energy Recovery Unit (model Danfoss-APP1.0/APM1.2). The RO membrane used is Hydraunatic SWC5-4040, from Lenntech company with an average salt rejection of 99.7%*.* Three modules were connected in a series arrangement (3 Pressure Vessels each equipped with a single module). Synthesized brackish water was prepared by dissolving industrial grade sodium chloride (sea salt) from El-Arish Governorate, Egypt. The salt chemical properties are presented in Table 1. Feedwater salinity was 10 mg/L, with an equivalent osmotic pressure equal to 8.61 bars. The osmotic pressure was calculated using Van’t Hoff relation. Permeate Total Dissolved Solids (TDS) was 192 mg/L, and brine TDS was 13.1 g/L as shown in Table 2.

**Table 1:** Chemical properties of the used salt.

Elements in the dry salt sample			
Sodium chloride	98.5%	Bicarbonate	4 × 10^–3^%
Moisture	0.23%	Iron	3 × 10^–6^%
Insoluble matter	0.02%	Copper	2 × 10^–6^%
Soluble matters	0.57%	Arsenate	2 × 10^–5^%
Calcium	0.0721%	Lead	2 × 10^–5^%
Magnesium	0.0722%	Mercury	5 × 10^–6^%
Sulphate	0.313%	KIO_3_	5.3 × 10^–3^%
Potassium	0.02%	Cadmium	8 × 10^–7^%

**Table 2:** Chemical properties of water samples used.

Element	pH	ppm	meq/L							mg/L			
			HCO_3_^-^	Cl^-^	SO_4_^–^	Ca^++^	Mg^++^	Na^+^	K^+^	NH4^+^	NO_3_^−^	Fe^++^	SAR
Feedwater	7.7	10,120	1.89	130	76.6	12.7	10.6	1.9	0.2	˂ 0.1	3.22	0.15	54.3
Brine	7.5	13,120	3.78	125	117.2	7.57	8.28	230	0.2	1.26	6.44	0.15	81.6
Permeate	8.1	192	1.31	1.5	0.2	0.4	0.26	2.3	-	0.42	8.96	0.14	4.11

*SAR* Sodium adsorption ratio, *HCO*_*3*_^*−*^ hydrogen carbonate ions, *SO*_*4*_^*–*^ sulfate ions, *Cl*^*−*^ chloride ions, *Ca*^*++*^ calcium ions, *Mg*^*++*^ magnesium ions, *Na*^*+*^ sodium ions, *K*^*+*^ potassium ions, *Fe*^*++*^ ferric, *NH*_*4*_^*+*^ ammonium ions, *NO*_*3*_^*−*^ nitrate.

The average pure water flux is 9.5 LMH and was calculated by dividing the permeate volume by the product of membrane surface area and time. Each batch run produced around 4 m^3^ of permeate, which was enough to irrigate the designated plant beds. The estimated average permeate recovery for the RO process is 22% and salt rejection exceeded 98.7%. The differential pressure between membrane inlet and outlet was equal to 1 bar, where membrane inlet pressure was 16 bars, and the outlet was 15 bars. The RO process operated at an average transmembrane pressure equal to 16 bars and an average permeate and brine flow rates equivalent to 3.49 and 12.41 Lpm, respectively. All experiment runs were performed at 25 °C.

### Plant materials and cultivation practice

Swiss chard bright lights (*Beta vulgaris* subsp. *cicia*) seeds were imported from Seed kingdom seed company in the USA. Seeds were sown in ¼ inch holes in a seed starting mix containing perlite and vermiculite and irrigated with a hand mist sprayer daily to keep the growing media always moist. Sowing was done on the 12th of November 2019, and seedlings were transplanted when they were 40 days old. Seedlings were transplanted into raised grow beds made of fiberglass material measuring 1.8 × 1.2 × 0.6 m for each of the four systems. The beds were raised off the ground by 0.5 m to allow drainage water from the bed to be collected and circulated back to the fish tank. Each bed was constructed with a drainage pipe at the bottom covered with a mesh net to prevent water blockage by the sand. Also, a 5 cm layer of small gravel was uniformly laid at the bottom of the beds to facilitate drainage, followed by sand with a height of 50 cm.

In the IAVS systems, plants were irrigated using manually punched diaphragm emitters, and the irrigation flow rate was controlled using small plastic valves at the start of every irrigation tube. Emitters were installed in drip tubing at a 30 cm distance as well the tubing lines were also placed 30 cm between each other. Seedlings were transplanted 5 cm away from the emitters at 30 cm between rows and 30 cm within the row. Since the water was pumped with submersible pumps to the grow beds, regulatory pressure valves were installed in between the pump and the main irrigation line, and then water flows through the emitters into the row furrows. Water would then saturate in the sand and eventually drain at the bottom into drainage tanks and pumped back to the fish tanks.

To maintain the water quality, two full cycles of water recirculation were run every day. Every irrigation cycle recirculated 25% of the fish tank, and complete drainage was allowed for a maximum of two hours. Plants were harvested upon reaching maturity for three cuts, except with the T1, which could not grow back after the second cut. Plants took 52 days from transplanting to reach the first cut, 20 days from cut 1 to cut 2, and as well 23 days from cut 2 to reach cut 3. Measurable crop parameters included plant height at harvesting/cutting, leaf area, number of leaves per plant, chlorophyll content, fresh weight per plant, and nutrient composition. Since the focus of SP is on the crops, fish were only measured to monitor their relative growth in terms of weight gained at harvesting/cutting time.

#### Measurement of crop parameters

Plants were cut 5 cm above the soil surface, and agronomical trait measurements from a representative sample of 12 plants per replicate were taken as follows.

Plant heights were taken using a foot ruler and averages determined. Leaf number was obtained as the number of leaves counted per plant and averages determined. Leaf area was calculated according to the equation reported by Yeshitila and Taye^16^.

$${\text{Leaf}} \, {\text{ Area }}\left( {{\text{cm}}^{{2}} } \right) = \, - {422}.{973} + { 22}.{752}0{\text{L }}\left( {{\text{cm}}} \right) \, + { 8}.{\text{31W }}\left( {{\text{cm}}} \right)$$

where L and W represent the leaf length and Leaf width respectively, − 422.973 is a constant relating to the shape of the leaf of Swiss chard developed by the author under citation.

Chlorophyll content was measured using MC-100 chlorophyll meter from Apogee Instruments, Inc, and data was expressed as SPAD averages. Fresh weight was measured using a digital weighing balance and data expressed as g/plant.

#### Sand test

Sand samples were obtained and sent for analysis at the Soils, Water and Environment Research Institute, Agricultural Research Center, Giza, Egypt. The Electrical conductivity (EC) values were measured from the sand paste extract; pH values were taken from sand suspensions at ratio of 1:2.5 as described by Estefan^17^. The available nitrogen in the sand sample was extracted using potassium chloride (KCl) as an extractable solution with the ratio of (5gm sand to 50 ml KCl) and determined using the micro- kjeldahl method. Available potassium was determined using a flame photometer, and the other elements in the sand sample were determined by using inductively coupled plasma (ICP) Spectrometry (model Ultima 2 JY Plasma)^18,19^. The physical and chemical characteristics of the used sand are presented in Table 3.

**Table 3:** (a): Chemical analysis of field sand samples, (b): Available macro, micronutrients, and heavy metals content of the sand samples.

(a)										
Sample	pH 1:2.5	EC ds/m	SP	Anions (meq./L)				Cations (meq./L)		
				HCO_3_	Cl	SO_4_	Cu	Mg	Na	K
T2	7.7	4.84	18	1.89	21.19	26.94	21.05	7.75	20.87	0.34
T3	7.3	4.52	18	2.12	16.95	32.68	22.37	8.07	20.87	0.45
T4	7.9	3.68	18	1.89	16.1	22.37	23.68	10.01	6.57	0.1

(b)										
Sample no.	(mg/kg)									
	N	P	K	Mn	Zn	Fe	Cu	Cd	Co	Pb
T2	52	0.03	37.2	2.4	0.21	2.98	0.33	* < 0.1	0.29	* < 1.5
T3	58	0.03	57.7	0.76	0.16	1.35	0.31	0.04	0.2	0.12
T4	53	0.02	29.8	0.19	0.46	1.23	0.29	0.07	0.33	* < 1.5

*Detection Limit (μg/kg).

*T2* October, *T3* Benu Suef location, *T4* Fayoum, *SP* Saturation percentage, *EC* electrical conductivity, *HCO*_*3*_ hydrogen carbonate, *SO*_*4*_ sulfate, *Cl* chloride, *Cu* copper, *Mg* magnesium, *Na* sodium, *K* potassium, *N* nitrogen, *Fe* iron, *Mn* manganese, *Pd* lead, *Co* cobalt, *P* phosphorus, *Zn* zinc, *Cd* cadmium.

#### Water analysis

Every 15 days, a measured amount of desalinated water was added to a standard mark of 850L in the fish tanks to compensate for the consumed amount of water in the system. Fish water quality parameters such as water temperature, pH, and dissolved oxygen (DO) was closely monitored using automated digital Nilebot technologies by Conative labs to fit the ideal required levels as reported by Somerville et al.^20^. In contrast, ammonia, nitrite, and nitrate were adjusted using an API test kit every week. These parameters' recorded values were as follows: water temperature ranged between 25 and 28 °C, DO range between 6–7 mg/L, and pH between 6.5 and 7.0. Ammonia levels were kept below 1 mg/L. Elements in water samples were determined according to EPA methods^18^ using inductively coupled plasma (ICP) Spectrometry (model Ultima 2 JY Plasma) as presented in Table 4.

**Table 4:** Water sample analysis for the different systems’ fish tanks and sump tanks.

Water sample		TDS (ppm)	pH	SAR	EC dS/m	Soluble Anions (meq./L)			Soluble Cations (meq./L)						NH_4_^+^ mg/L	NO_3_^-^ mg/L	P mg/L
						HCO_3_^−^	SO4^2−^	Cl^−^	Ca^2+^	Mg^2+^	Na^+^	K^+^	Fe^3+^	Mn^2+^			
T1	Fish tank	403	7.4	1.4	0.63	2.16	2.14	2	2.01	1.7	1.9	0.69	0.15	< 0.5	< 1.0	10.2	< 1.5
	Sump tank	243	7.5	0.57	0.38	1.08	1.12	1.6	1.5	0.89	1.4	-	0.14	< 0.5	< 1.0	5.3	< 1.5
T2	Fish tank	1459	6.8	1.32	2.28	4.5	16.3	2	10	8.47	4	0.33	0.14	< 0.5	< 1.0	9.6	< 1.5
	Sump tank	1766	7.3	1.5	2.76	5.4	19.2	3	14.1	8.2	5	0.26	0.17	< 0.5	1.9	4.13	< 1.5
T3	Fish tank	1638	7	0.16	2.56	5.4	18.2	2	13.4	8.5	3.5	0.23	0.15	< 0.5	1.82	8.9	< 1.5
	Sump tank	1670	7.3	0.14	2.61	5.4	17.7	3	12.3	10	3.6	0.18	0.19	< 0.5	< 1.0	5.25	< 1.5
T4	Fish tank	1709	6.8	0.28	2.67	4.05	20.65	2	17	16.9	4.4	0.42	0.19	0.07	< 1.0	7.8	< 1.5
	Sump tank	1766	7.3	0.21	2.76	4.05	19.55	4	12.1	11.2	3.9	0.39	0.18	< 0.5	< 1.0	5.7	< 1.5

*TDS* total dissolved solids, *SAR* Sodium adsorption ratio, *EC* electrical conductivity, *HCO*_*3*_^*−*^ hydrogen carbonate ions, *SO*_*4*_^*2−*^ sulfate ions, *Cl*^*−*^ chloride ions, *Ca*^*2+*^ calcium ions, *Mg*^*2+*^ magnesium ions, *Na*^*+*^ sodium ions, *K*^*+*^ potassium ions, *Fe*^*2+*^ ferric, *Mn*^*2+*^ manganese ions, *NH*_*4*_^*+*^ ammonium ions, *NO*_*3*_^*−*^ nitrate, *P* phosphorus.

#### Nutritive composition analysis

According to Official methods of analysis from the association of official analytical chemists (A.O.A.C) (1990), moisture content and Vitamin C were determined. Vitamin A was determined according to the procedures described by Aremu and Nweze^21^. Briefly, 100 g of the sample were homogenized, from which 1 g was obtained and soaked in 5 mL methanol for two hours at room temperature in the dark for complete extraction of a pro-vitamin A carotenoid, β-carotene. Separation of the β-carotene layer was achieved through the addition of hexane to the sample, and moisture was removed using sodium sulphonate. The absorbance of the layer was measured at 436 nm using hexane as a blank. β-carotene was calculated using the formula:

$$\beta {\text{-carotene }}\left( {{\mu g}/{1}00{\text{ g}}} \right) \, = {\text{ Absorbance }}\left( {\text{436 nm}} \right) \, \times {\text{ V }} \times {\text{ D }} \times { 1}00 \, \times { 1}00/{\text{W }} \times {\text{ Y}}$$

where: V = total volume of the extract; D = Dilution factor; W = Sample weight; Y = Percentage dry matter content of the sample.

Vitamin A was then determined according to the concept of Retinol Equivalent (RE) of the β-carotene content of the vegetables using the standard conversion formula. Total hydrolyzable carbohydrates were determined as glucose using phenol–sulfuric acid reagent as described by Michel^22^.

Vitamin C content was determined using dichlorophenol indophenol reagent. As such, 10 g of fresh leaf tissues, were crushed using a motor and pestle in the presence of 10 ml metaphosphoric acid 6% (Merck). This was followed by centrifugation at 4000×*g* for 5 min at 4 °C. Five mL of the supernatant were transferred into an Erlenmeyer flask, and 20 mL of 3% metaphosphoric acid were added. The extract was titrated by dichlorophenol indophenol (Sigma-Aldrich) until a rose color was observed. Vitamin C (mg/100 g FW) was then calculated and based on the standard curve of l-Ascorbic acid (Merck) concentrations.

For the determination of protein and mineral content, 0.5 g of dried samples were digested using sulfuric acid (H_2_SO_4_) and hydrogen peroxide (H_2_O_2_) as described by Cottenie^23^. From the extracted sample, the following minerals were determined:

Nitrogen was determined according to the procedures described by Plummer^24^. Briefly, 5 mL of the digestive solution was distilled with 10 mL of sodium hydroxide (NaOH) for 10 min to obtain ammonia. Back titration was then used to determine the amount of nitrogen present in ammonia. Protein content was calculated by multiplying total nitrogen by 6.25 according to methods of AOAC^25^.

Phosphorus content was determined calorimetrically (660 nm) according to the procedures described by Jackson^26^. Potassium, Calcium, and Sodium were determined against a standard using a flame-photometer (JEN way flame photometer) as described by Piper^27^. Magnesium (Mg), Copper (Cu), Manganese (Mn), Zinc (Zn), and Iron (Fe) content were determined using Atomic Absorption Spectrophotometer, Pyeunican SP1900, according to methods described by Liu^28^.

The moisture percentage of leaf samples was determined by weighing the fresh weight for each sample (Fw), then dried for 72 h at 80 °C. The dry matter weight was record as Dw. The leaf water content was then calculated as the following:

$${\text{Moisture}}\;{\text{ content }}\left( \% \right) \, = \, \left( {{\text{Fw}} - {\text{Dw}}} \right) \, /{\text{ Fw}} * {1}00$$

### Statistical analysis

Statistical comparisons among means of more than two groups were performed with analysis of variance (ANOVA) using SPSS V22, and the difference in means was analyzed by Tukey’s test at α = 0.05. Statistical differences were considered significant at P ≤ 0.05 in triplicates and data expressed as mean ± S.D.

### Plant material

All plant materials and related procedures in this study were done in accordance with the guidelines of the Institutional Review Board of the American University in Cairo and the Ministry of Agriculture and Land Reclamation in Egypt.

### Ethics approval

This study followed the guidelines and approval of Committee of Animal Welfare and Research Ethics, Faculty of Agriculture, Kafrelsheikh University, Egypt.

## Results

### Effect of different treatments on vegetative growth parameters of Swiss chard

**Figure 2:**
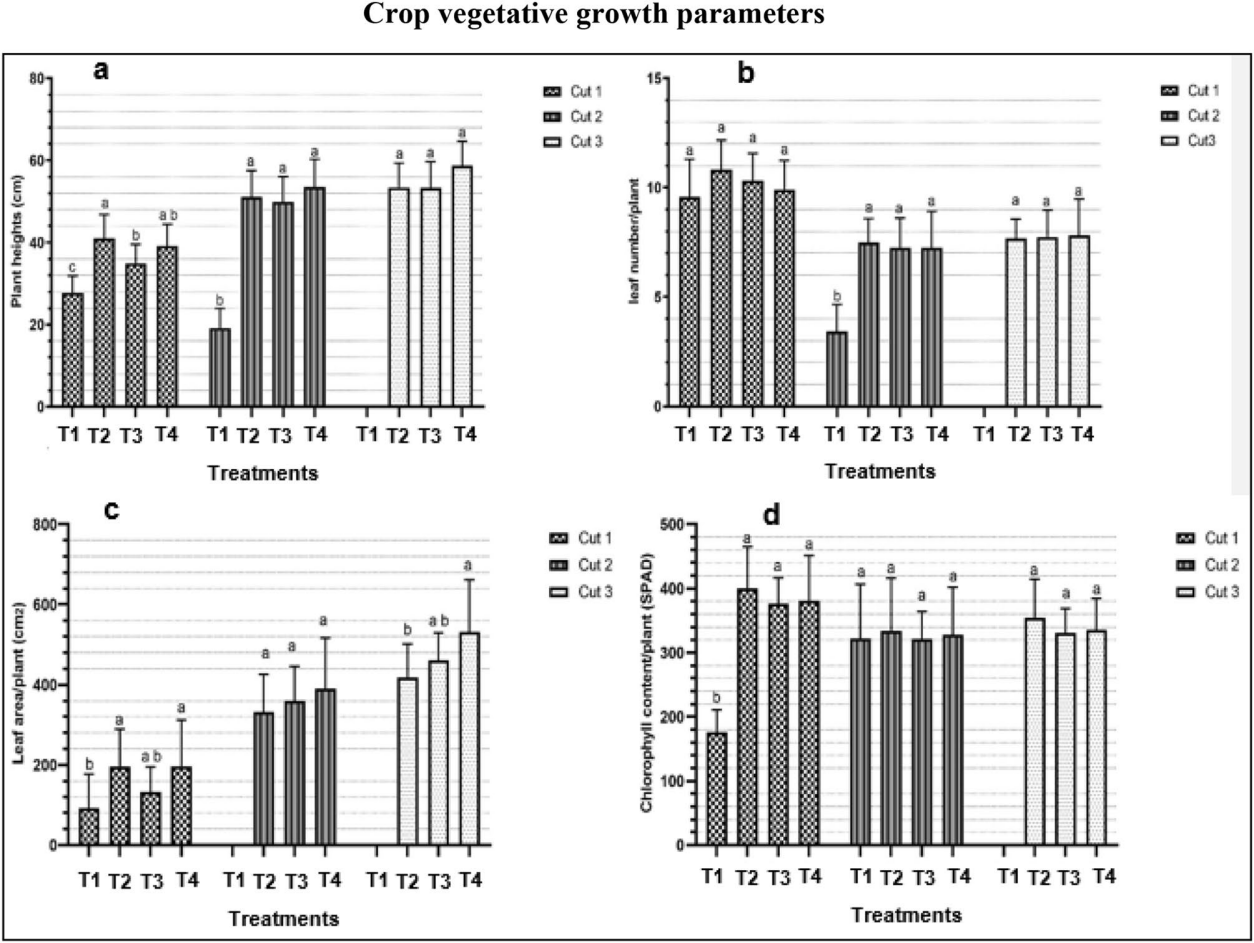
Effect of different treatments on vegetative growth; (**a**) Plant height; (**b**) Leaf number; (**c**) Leaf area, and (**d**) chlorophyll content at different cut numbers. Data are expressed as mean ± SD (n = 12). Error bars represent the standard deviation. Bar columns within the same cut number having the same letter at the top are not significantly different (P > 0.05). *T1* Deep water culture, *T2* October, *T3* Beni suef, *T4* Fayoum.

The effect of different treatments on plant height, leaf number, leaf area, and chlorophyll content at different cut numbers is shown in Fig. 2. Results from cut 1 show that plants in the T1 significantly recorded the lowest plant heights (27.83 cm) compared to other treatments (*P* < 0.0001). Likewise, T2 significantly had a higher plant height (41.05 cm) compared to T3 (34.93 cm) (*P* < 0.0001). No significant difference in plant height was noted between T2 and T4 during cut 1. In cut 2, still, T1 significantly recorded the lowest plant height (19.18 cm) compared to T2, T3, and T4 (51.2 cm, 49.9 cm, and 53.5 cm, respectively) (*P* < 0.0001). However, the results of the third cut showed no significant difference in plant height among the three treatments T2, T3, and T4 (Fig. 2a). A significant interaction between treatments and the cut number was observed with respect to plant heights (*P* < 0.0001). Generally, plant heights increased with the increase in the number of cuts across treatments except for T1.

Data on the effect of different treatments on leaf numbers are shown in Fig. 2b. The first and third cut results indicate no significant difference in leaf number among all treatments even though T2 recorded the highest with 10.8 leaves/plant. For the second cut, however, plants in the T1 significantly had the lowest leaf number (3 leaves/plant) compared to other treatments (*P* < 0.0001). Furthermore, there was a statistically significant interaction between treatments and cut number, *P* < 0.0001. Overall, leaf number decreased across all treatments between cut one and two but was maintained at almost eight leaves per plant in both the second and third cut.

Results on the effect of different treatments on leaf area are presented in Fig. 2c. For cut one, leaf area varied across different treatments, and plants in the T1 significantly had a smaller leaf area (92.92 cm^2^) compared to T2 and T4 (195.30 cm^2^ and 196.95 cm^2^, respectively) (*P* < 0.0001). In the second cut, T2, T3, and T4 treatments showed no significant difference in their leaf area per plant. However, data obtained in the third cut indicated that plants in the T2 treatment significantly had a smaller leaf area (417.63 cm^2^) compared to T4 (530.85 cm^2^) except for T3 (*P* < 0.05). No significant interaction was noted between treatments and cut number regarding leaf area. Overall, it was noted that leaf area significantly increased with the increase in the number of cuts across all treatments.

Figure 2d shows the effect of different treatments on the chlorophyll content of plant leaves. For cut one, plants in the T1 significantly recorded the lowest chlorophyll content (176.33 SPAD) compared to T2, T3, and T4 (399.70, 375.95, and 380.81 SPAD, respectively) (*P* < 0.0001). However, no significant difference in chlorophyll content of plant leaves was noted across treatments for both cut 2 and cut 3. A statistically significant interaction between treatments and the cut number was noted (*P* < 0.0001). Generally, the chlorophyll content decreased across all treatments in the first and second cut but slightly increased during the third cut.

### Effect of different treatments on biomass yield of Swiss chard

**Figure 3:**
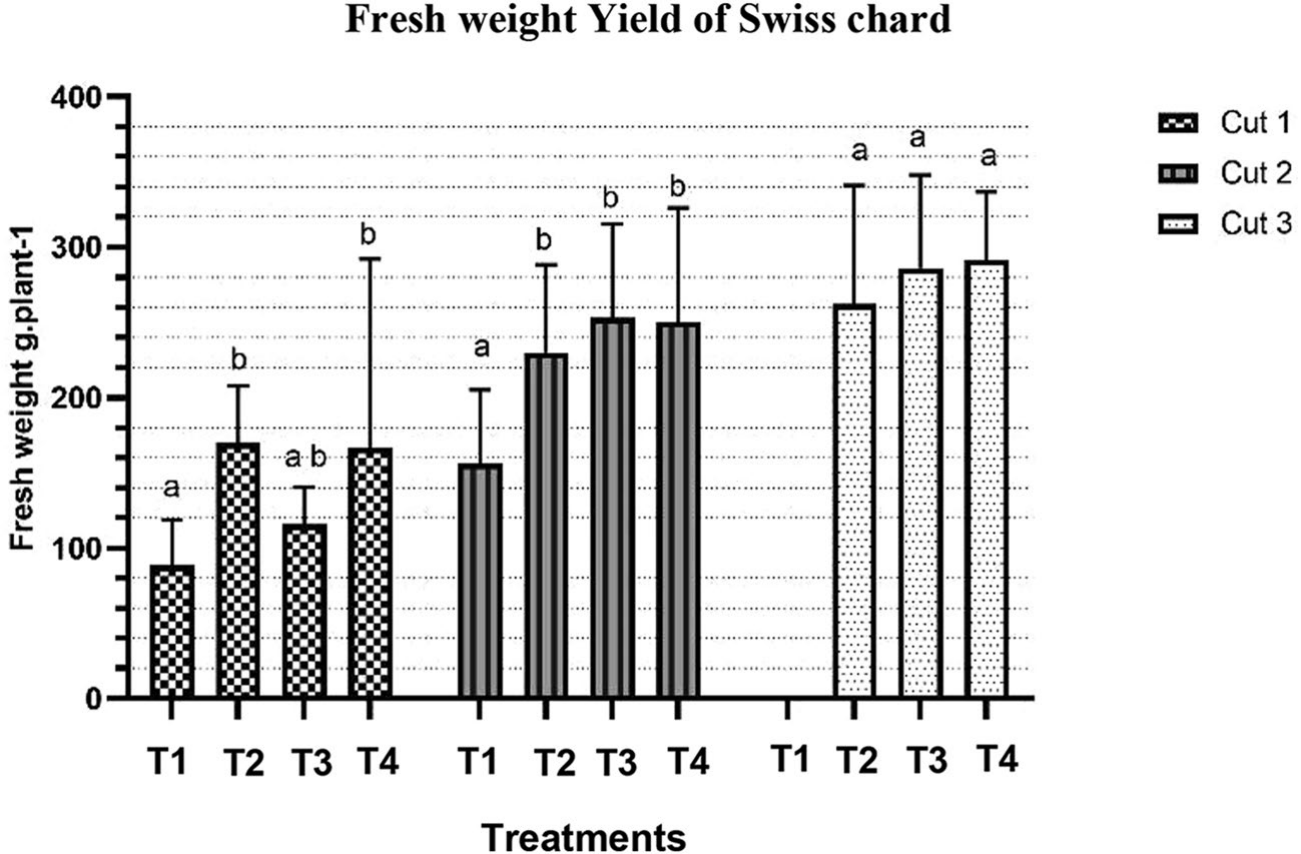
Effect of different treatments on fresh weight of Swiss chard at different cut numbers. Data are expressed as mean ± SD (n = 24). Error bars represent the standard deviation. Bar columns within the same cut number having the same letter at the top are not significantly different (*P* > 0.05). *T1* Deep water culture, *T2* October, *T3* Beni suef, *T4* Fayoum.

The effect of different treatments on the fresh weight of Swiss chard at different cut numbers is presented in Fig. 3. There was a variation in fresh weights across all treatments in the first cut. The data shows that T1 significantly had the lowest fresh weight (88.29 g/plant) compared to T2 and T4 (169.75 and 166.38 g/plant respectively) (*P* < 0.0001). However, no significant difference in the average fresh weight was noted among T2, T3, and T4, respectively. Likewise, results of the second cut show that T1 significantly had the lowest fresh weight (156.13 g/plant) compared to other treatments (*P* < 0.0001). No significant difference in the average fresh weight was noted among T2, T3, and T4 in the third cut. There was a significant interaction between treatments and the cut number, *P* < 0.05. Overall, the average fresh weight increased with the increasing number of cuts across all treatments.

Results of the average yield for cut 1 indicate that T1 had the lowest yield (14.13 ton/ha) compared to T2 (27.16 ton/ha), T3 (18.56 ton/ha), and T4 (26.62 ton/ha). For cut 2, T1 had the lowest yield (24.96 ton/ha) compared to T2 (36.81 ton/ha), T3 (40.49 ton/ha), and T4 (41.63 ton/ha). Results for cut 3 shows that T2 had a slightly lower yield (41.99 ton/ha) compared to T3 (45.77 ton/ha) and T4 (46.56 ton/ha).

### Water consumption of Swiss chard grown under different sand media based IVAS system

**Figure 4:**
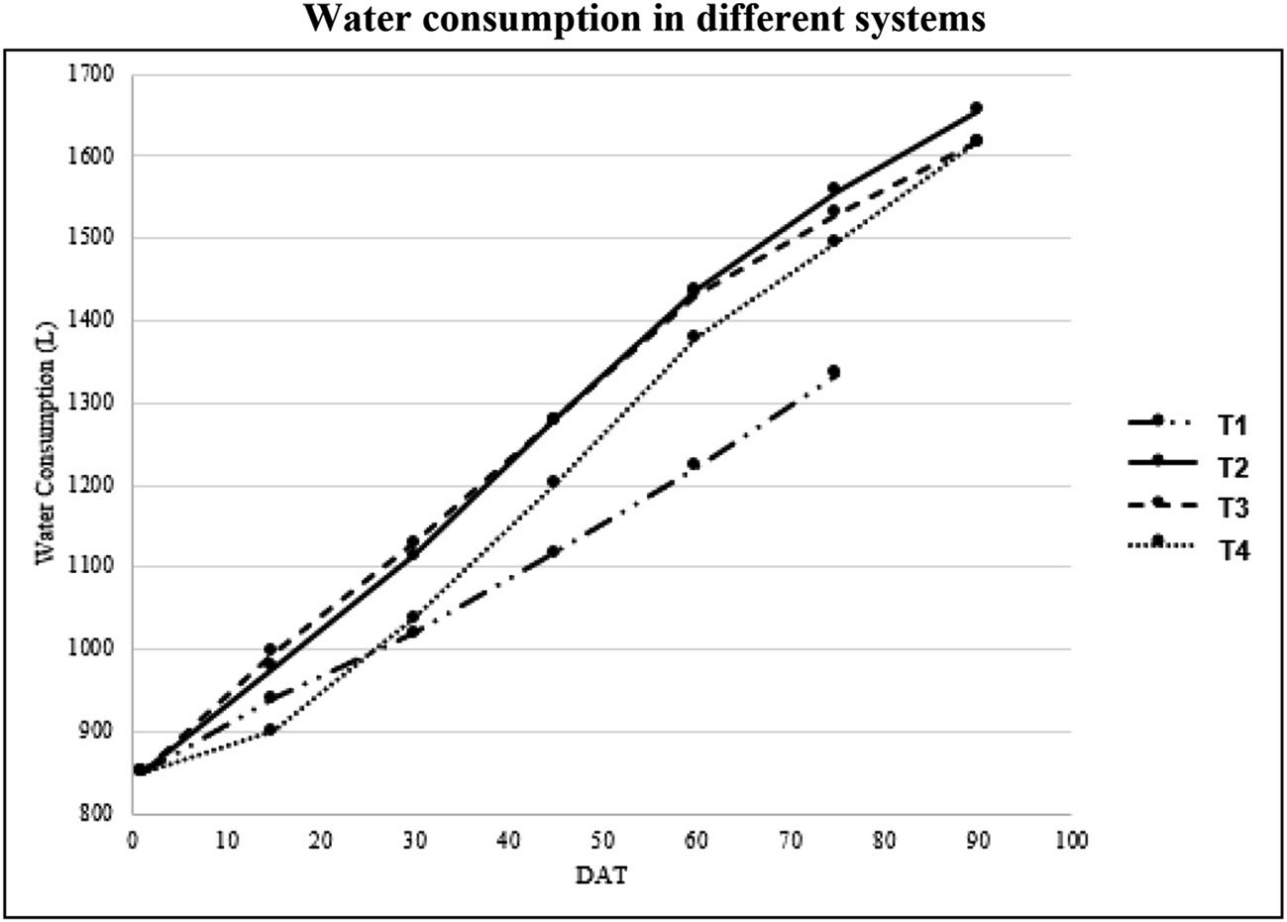
Water consumption during the three cuts. *T1* Deep water culture, *T2* October, *T3* Beni suef, *T4* Fayoum, *DAT* Days after transplanting.

The water consumption of Swiss chard grown under different sand media based IVAS systems was calculated. Figure 4 shows the cumulative water consumption in all systems during all the three cuts. Since the fish water quantity was fixed at 850 L, during the first cut, T1 used the least amount of water (372 L) followed by T3 (529 L), T2 (582 L), and T4 (587 L), respectively. In the second cut, T1 still used the least amount of water (113 L) followed by T3 (116 L), T2 (98 L), and T4 (120 L), respectively. In the third cut, T2 and T4 used a lower amount of water (87 L and 99 L, respectively) compared to T3 (120 L). Considering crop water consumption during the three cuts or period of 95 days of experimentation, except for T1, which was only harvested for two cuts; T1 consumed 1.56 L/m^2^/day, T2 and T3 with 1.87 L/m^2^/day, and T4 with 1.96 L/m^2^/day.

### Effect of different treatments on the nutritive composition of Swiss chard

In all cut numbers of all treatment groups, the moisture content (%) ranged between 92 to 93% thus indicating no significant difference among all treatments. However, data shows that cut three significantly had a lower moisture (%) compared to cut one and two (P < 0.0001). A highly significant interaction between treatments and the cut number was noted (P < 0.0001).

The protein content of plants sampled from all the treatment groups ranged from 11.84 to 16.79 mg/100 g DW in cut one. No significant difference was noted among all treatment groups. For cut two, plant samples obtained from the T4 treatment group significantly had the highest protein content (18.72 mg/100 g DW) compared to T3 (13.67 mg/100 g DW) and T1 (13.35 mg/100 g DW) (P < 0.05). No significant difference was noted in protein percentage from all plant samples obtained from all treatment groups (T2, T3, and T4) in the third cut. However, a highly significant interaction between treatments and the cut number was noted (P < 0.0001). Overall, cut three had the least protein content than cut one and two, which was significant at P < 0.05.

Data obtained for total carbohydrate content indicates no significant difference among all treatments at different cut numbers. However, treatments combined, the third cut significantly had the lowest total carbohydrate content compared to other cuts (P < 0.05). Also, there was a significant interaction between treatments and the cut number (P < 0.0001).

The vitamin content of plant samples was assessed at different cuts too. Results of cut one indicate that plant samples obtained from the T2 treatment group significantly contained higher vitamin A (2.36 mg/100 g FW) and vitamin C (19.55 mg/100 g FW) compared to those obtained from T4 and T1, and T4 treatments groups respectively (P < 0.05). However, no significant difference in vitamin A and C was noted for cut two and cut three among all treatment groups. Overall, treatments combined, no significant difference in vitamin A content were noted among all cut numbers. Cut one significantly had a lower vitamin C content for vitamin C content than cut two (P < 0.05).

The mineral composition of plant samples (Table 5) shows no significant difference in Fe, Mn, and Cu contents among all treatment groups in all cut numbers. However, Mg content of plant samples obtained from the T1 treatment group in cut one was significantly higher (1841.36 mg/100 g DW) compared to T4, T3, and T2 (1151.28, 959.07, and 863.66 mg/100 g DW respectively) (P < 0.05). No significant difference was noted among all treatment groups in cut two and cut three. The Zn content of plant samples obtained from T3 in cut one was significantly lower (99.08 mg/100 g DW) than T1, T2, and T4 (109.95, 103.78, and 102.27 mg/100 g DW respectively). No significant difference was noted among all treatment groups in cut two and cut three.

**Table 5:** Nutritive composition of Swiss chard for the three cuts.

Treatments	Moisture (%)	Protein (g/100 g DW)	Total carbs (g/100 g DW)	Vit. A (mg/100 g FW)	Vit. C (mg/100 g FW)	Fe (mg /100 g DW)	Mg (mg/100 g DW)	Mn (mg/100 g DW)	Ca (mg/100 g DW)	Cu (mg/100 g DW)	Zn (mg/100 g DW)
**CUT. no. 1**											
T1	92.95^a^ ± 1.31	11.84^a^ ± 4.45	41.03^a^ ± 9.33	1.30^b^ ± 0.81	13.98^ab^ ± 4.51	294.79^a^ ± 70.66	1841.36^a^ ± 726.82	5.59^a^ ± 2.25	795.20^ab^ ± 213.01	4.07^a^ ± 1.17	109.95^a^ ± 2.42
T2	92.22^a^ ± 1.13	15.03^a^ ± 2.77	42.77^a^ ± 8.72	2.36^a^ ± 0.63	19.55^a^ ± 8.20	404.99^a^ ± 29.99	863.66^b^ ± 179.88	6.35^a^ ± 4.87	1134.75^a^ ± 285.58	4.29^a^ ± 3.29	103.78^ab^ ± 5.78
T3	92.57^a^ ± 1.13	16.79^a^ ± 3.21	45.76^a^ ± 3.93	1.69^ab^ ± 0.59	14.82^ab^ ± 3.31	515.47^a^ ± 277.45	959.07^b^ ± 371.00	6.99^a^ ± 3.51	695.72^b^ ± 119.59	4.48^a^ ± 1.83	99.08^b^ ± 5.50
T4	92.91^a^ ± 1.47	15.11^a^ ± 3.00	38.35^a^ ± 7.55	1.34^b^ ± 0.18	11.22^b^ ± 2.46	472.00^a^ ± 135.26	1151.28^b^ ± 174.17	6.99^a^ ± 2.47	747.18^b^ ± 201.64	4.67^a^ ± 1.58	102.27^ab^ ± 6.20
**CUT. no. 2**											
T1	92.57^a^ ± 0.83	13.35^b^ ± 4.50	36.56^a^ ± 4.50	1.39^a^ ± 0.16	16.18^a^ ± 2.04	197.16^a^ ± 12.94	575.41^a^ ± 73.98	6.89^a^ ± 2.21	272.82^a^ ± 82.15	5.08^a^ ± 1.23	18.12^a^ ± 3.15
T2	92.22^a^ ± 1.13	15.39^ab^ ± 2.86	42.97^a^ ± 12.62	1.41^a^ ± 0.22	17.39^a^ ± 3.60	246.69^a^ ± 53.79	643.68^a^ ± 217.69	11.43^a^ ± 10.14	356.18^a^ ± 467.10	6.48^a^ ± 2.76	18.97^a^ ± 3.45
T3	92.57^a^ ± 1.13	13.67^b^ ± 2.33	34.27^a^ ± 8.11	1.62^a^ ± 0.31	23.33^a^ ± 9.90	309.51^a^ ± 96.75	654.37^a^ ± 237.51	7.49^a^ ± 2.32	469.65^a^ ± 178.05	5.13^a^ ± 1.68	21.46^a^ ± 1.34
T4	92.66^a^ ± 2.17	18.72^a^ ± 3.36	39.85^a^ ± 4.39	1.85^a^ ± 0.45	18.72^a^ ± 3.01	238.84^a^ ± 90.67	772.71^a^ ± 217.44	7.70^a^ ± 3.47	399.67^a^ ± 151.52	5.58^a^ ± 1.80	20.34^a^ ± 1.50
**CUT. no. 3**											
T2	92.14^a^ ± 0.93	16.39^a^ ± 2.19	42.06^a^ ± 13.05	1.61^a^ ± 0.80	28.83^a^ ± 5.48	256.64^a^ ± 17.35	686.88^a^ ± 70.08	10.34^a^ ± 2.01	263.56^c^ ± 40.54	6.61^a^ ± 0.81	21.39^a^ ± 1.45
T3	92.76^a^ ± 1.71	14.89^a^ ± 1.98	36.40^a^ ± 4.28	2.09^a^ ± 0.28	19.80^a^ ± 6.92	242.52^a^ ± 11.05	636.29^a^ ± 70.99	8.77^a^ ± 2.32	344.52^ab^ ± 58.81	5.32^a^ ± 1.18	20.21^a^ ± 0.92
T4	93.54^a^ ± 1.49	16.15^a^ ± 3.38	35.26^a^ ± 5.57	2.07^a^ ± 0.31	24.89^a^ ± 10.15	240.49^a^ ± 21.07	704.66^a^ ± 79.45	9.20^a^ ± 3.46	353.08^a^ ± 114.88	6.04^a^ ± 1.15	20.04^a^ ± 1.76

Nutritive composition values are expressed as mean ± SD (n = 6). Values within the same column in the same cut number having different superscript letters are significantly different at *P* < 0.05 (Tukey test). Treatments are; *T1* Deep water culture, *T2* 6th October, *T3* Benu Suef and *T4* Fayoum. *Carbs* carbohydrates, *Vit. A* Vitamin A, *Vit. C* Vitamin C, *Fe* Iron, *Mg* Magnesium, *Mn* Manganese, *Ca* Calcium, *Cu* Copper, *Zn* Zinc.

Results for Ca content show that plant samples obtained from the T2 treatment group in cut one significantly had a higher Ca content (1134.75 mg/100 g DW) compared to T4 and T3 (747.18 and 695.72 mg/100 g DW, respectively) (P < 0.05). However, data obtained in cut three indicates that plant samples obtained from the T4 treatment group significantly contained higher Ca content (353.08 mg/100 g DW) followed by T3 (344.52 mg/100 g DW) and T2 (263.56 mg/100 g DW) respectively (P < 0.05). There was a significant interaction between treatments and the cut number (P < 0.05) except for Fe. Overall, treatments combined, the mineral composition (Fe, Mg, Ca, and Zn) in cut one was significantly higher compared to cut two and cut three (P < 0.05). However, no significant difference was noted in Mn and Cu contents in all cut numbers.

## Discussion

According to some authors^29–31^, the primary sources of nutrients in aquaponics or sandponics are the water source added (containing Mg, Ca, S), uneaten fish feed, and fish waste. The fish effluents contain a significant level of nitrogen and phosphorous, although with fewer quantities of microelements. Both macro and microelements are vital for plant’s growth in varying amounts. The main essential elements include N, K, Ca, Mg, P, S, and the microelements Fe, Mn, B, Zn, Cu, and Mo^20,32^. The primary required nutrient for the system is nitrates. The exact concentrations of these nutrients in our effluent water are presented in Table 4. Although all of these nutrients exist in solid fish waste, some nutrients, especially Ca, K, and Fe may be limited in aquaponics and may result in plant nutritional deficiencies^32,33^. Our results for nitrates concentration conform to a range of 5–150 mg/L recommended by FAO^20^.

Yang and Kim^34^ have recently experimented on the yield and nutrient management in aquaponics using leafy vegetable crops, including Swiss chard. They reported a maximum plant height of 27 cm. This study results showed that edible Swiss chard could reach more than 53 cm height in the second cut, almost twice the height recorded by the authors above. In this current study, Yang and Kim also reported approximately the same number of leaves obtained per plant. The discrepancy in results could be attributed to the differences in growth media used in both experiments. In our study, the different sand-based IVAS systems supplemented plants with Ca, Mg, Na, and S nutrients which enhanced their growth in contrast to the T1 system solely used in the study conducted by Yang and Kim^34^. However, our study's plants grown in the DWC system wilted during the third cut, probably due to very low EC values and lack of enough nutrients. Previous studies have shown that very low EC limits plant growth due to nutrient deficiency^35,36^.

Crop water consumption directly affects the vegetative performance and growth of the plants. Less vegetative structure can also mean less water usage. Since plants in the T1 are partially submerged in the effluent water at all times, not like in sandponic systems, they consumed less water of 1.56 L/m^2^/day than T2 and T3 with 1.87 L/m^2^/day, and T4 with 1.96 L/m^2^/day. Water consumption was significantly different between DWC and SP systems.

Regarding biomass production in an aquaponics experiment with Swiss chard, Kaburagi et al.^37^ investigated growing Swiss chard (*Beta vulgaris* L. spp. *Cicla*) using saline fish wastewater with micronutrient supplementation. Their results reported approximately 68, 42, and 18 g/plant leaves fresh weight during the first, second, and third cuts, respectively, using fish wastewater and micronutrient supplements. Irrigating plants with fish wastewater alone reported 65, 25, and 10 g/plant of fresh leaf weight during the first, second, and third cut. Their total yields amounted to 138 g/plant and 100 g/plant for fish wastewater plus micronutrients and fish wastewater alone. In our study, however, we recorded higher yields from the range of 88–170 g/plant for the first cut, then 156–255 g/plant and 260–280 g/plant in the third cut without supplementary application of micronutrients. The difference in results could be attributed to the growth media used, type of fish, and other parameters such as feeding rate for fish, etc. It’s hypothesized that the sand-based IVAS systems used in our study provided micronutrients and a desirable EC that enhanced nutrient uptake in plants, improving their growth.

Mineral elements are significantly essential for any plant’s growth. Calcium is the plant nutrient most often associated with tissue firmness. This is due to its ability to form cross-linkages with pectins by the ionic association between C’6 carboxyl groups of intra and inter galacturonosyl residues^38^. In principle, high calcium levels maintain tissue integrity and increase tissue elasticity rather than tissue rigidity. The increasing leaf area and total fresh weights recorded in T2, T3, and T4 SP treatments could be attributed to high calcium contents in the irrigation water compared to those grown in T1. Likewise, previous studies have shown that a high magnesium supply and EC increase tissue firmness^39,40^. This study shows that the magnesium supply and EC of irrigation water in SP treatments were higher than the DWC. This could also explain larger leaf areas and higher fresh weights in plants grown in SP treatments than in DWC.

On the other hand, magnesium is an important nutrient required for plant growth. It is not only the central core of the chlorophyll molecule in plant tissues but also aids in the activation of specific enzymes. Magnesium deficiency in plants results in stunted growth^41^. In this study, we recorded higher quantities of magnesium (575–184.36 mg/100 g DW) which was higher than that reported in previous studies^42,43^.

Microelements such as Fe, Cu, Mn, and Zn play a vital role in redox processes, and cofactors activate approximately 35 different enzymes^44^. Concentrations of Fe and Zn were within those of Swiss chard reported by Bozokalfa et al.^45^ except for Cu. Likewise, this study's Cu and Mn contents were lower than those reported in previous studies^43,46^. This could be attributed to the difference in cultivation practice and variety of the plant.

Vitamin C (ascorbic acid and dehydroascorbic acid) is one of the most important quality parameters of fruits and vegetable crops. It is an essential vitamin for humans, and 90% of it is obtained from fruits and vegetables. However, its content in plant tissues depends on the EC of irrigation water and levels of antioxidant enzyme activity. Ascorbic acid is one of the major antioxidants which could be stimulated under abiotic stress. Likewise, antioxidant enzyme activity is important for determining whether the plant is suffering from biotic or abiotic stress^35^. This study observed an increase in vitamin C content with an increasing cut number. Therefore, we anticipate that a combination of high EC (2.6–2.8 ds/m) and plant cuts increased plant tissue stress levels, resulting in high antioxidant enzyme activity and increasing vitamin C content. Similar results have been reported on tomatoes^38^ and *Brassica campestris* L. ssp. *Chinensis*^35^. Moreover, the vitamin C concentrations in this study ranged from 11.22 to 28.88 mg/100 g fresh weight, which is lower to those reported by Ivanović et al.^46^ and higher than that reported by Daiss et al.^47^ and Agüero et al.^48^. We suggest that the difference in results could be attributed to differences in cultivation practices and plant species.

Beta carotene is a precursor for vitamin A and one of the most important vitamins for human health. Vegetables from the *Brassicaceae* family contain the highest beta carotene amounts, ranging from 0.50 to 19.60 mg/100 g FW^49^. In our study, vitamin A ranged from 1.30 to 2.36 mg/100 FW, higher than that reported by Mzoughi et al.^44^. However, our results are not in agreement with those reported by Ivanović et al.^46^. They reported higher amounts of beta carotene (4.41 to 13.40 mg/100 g fresh weight). Likewise, Reif et al.^49^ reported higher amounts of beta carotene (6.82 mg/100 g FW) in cv. Carrot bolero of the *Daucus carota* L. Swiss chard species. The difference could be attributed to plant species or cultivars used, cultivation practices, and chemicals used in the extraction protocol. We compared the values for the Swiss chard variety used in this study with those referenced by the USDA data (3.64 mg/100 g FW) and found a minimal difference hence making this variety a good source of vitamin A.

High levels of soluble sugars are a desirable parameter in terms of food quality. Soluble sugar contents are influenced by several factors such as salinity, EC of irrigation water, and cutting time point. In this study, high EC values (2.6–2.8 dm/s) of irrigation water were noted in the studied SP treatments, which could cause declining contents of total carbohydrates. Similar results have been previously reported by Ding et al.^35^. High EC increases the respiration rate of tissues which might reduce sugar content^50^. The quantified total proteins in this study were higher than those earlier reported by Daiss et al.^47^ and lower than those reported by Ivanovic et al.^46^. This could be attributed to differences in variety, cultivation practices, and extraction methods. In this study, we reported similar crop water contents ranging from 92.14 to 93.54%, which was in the same range as that reported by Moreira et al.^51^ and Daiss et al.^47^.

## Conclusion

This study found that sandponics can be an alternative way to produce an increased crop yield in poor sand soils if the soil chemical composition does no harm to the fish. Sandponics uses synergies since the fish wastewater acts as a nutrient source to the crops, and sand acts as a bio-filter to clean the fish water. Sandponics is a promising sustainable crop production technique, efficient and low-risk application. This experiment found that sourcing good quality sand with low levels of carbonates, EC, Na, K, heavy metals and low levels of salts is essential for an efficient functionality of the sandponics system. Additionally, a neutral pH, sand grain size and good drainage are other key factors for an efficient system. Sand quality composition like that from T4 and T3 would be a great sand component/unit of the sandponics system. It drains efficiently, low levels of heavy metals plus carbonates. The study recommends that T4 and T3 sand types would be able to produce sufficient crop yields in terms of biomass and nutritive composition. As compared to DWC, sandponics also minimize water usage to as low as 1.96 L/m^2^/day. Further research is needed to investigate similar sand qualities with different crops and different fish types (Supplementary Tables 1 and 2).

## Supplementary Information

Supplementary Tables.

## Data Availability

The datasets generated during and/or analyzed during the current study are available from the corresponding author on reasonable request.
