# Supplementary material for: A sandponics comparative study investigating different sand media based integrated aqua vegeculture systems using desalinated water
Source: Sci Rep. 2022 Jun 30;12:11093. doi: 10.1038/s41598-022-15291-7 (PMC9247079; doi:10.1038/s41598-022-15291-7)
Supplement: Supplementary file 1 — Supplementary Tables. [file 41598_2022_15291_MOESM1_ESM.docx]

Supplemental Materials:

**Table 1. Two-way ANOVA (P value) for the data represented in Fig 2 & 3**

| Two-way ANOVA (*P* value) | Plant height (cm) | Leaf number/plant | Leaf area (cm^2^) | Chlorophyll content (SPAD) | Fresh weight (g.plant-1) |
| --- | --- | --- | --- | --- | --- |
| Cut number | 0.0001 | 0.0001 | 0.0001 | ------ | 0.0001 |
| Treatment | 0.0001 | 0.0001 | 0.05 | 0.0001 | 0.0001 |
| Cut number × Treatments | 0.0001 | 0.0001 | ------ | 0.0001 | 0.05 |

*Two-way ANOVA (*P* value) was calculated if significances existed.

**Table 2. Two-way ANOVA (P value) for the data represented in Table 6**

| Two-way ANOVA (*P* value) | Moisture  (%) | Protein  (g/100 g DW) | Total Carbs  (g/100 g DW) | Vit. A  (mg/100 g FW) | Vit. C  (mg/100 g FW) | Fe  (mg /100 g DW) | Mg  (mg /100 g DW) | Mn  (mg /100 g DW) | Ca  (mg /100 g DW) | Cu  (mg /100 g DW) | Zn  (mg /100 g DW) |
| --- | --- | --- | --- | --- | --- | --- | --- | --- | --- | --- | --- |
| Cut. No. | 0.0001 | 0.0001 | 0.0001 | ------ | 0.031 | 0.0001 | 0.0001 | ------ | 0.0001 | 0.041 | 0.0001 |
| Treatment | 0.0001 | 0.0001 | 0.0001 | 0.0001 | 0.0001 | 0.0001 | ------ | 0.002 | 0.011 | 0.0001 | 0.0001 |
| Cut. No. × Treatments | 0.0001 | 0.0001 | 0.0001 | 0.0001 | 0.0001 | ------ | 0.0001 | 0.043 | 0.005 | 0.0001 | 0.0001 |

*Two-way ANOVA (*P* value) was calculated if significances existed
